# Nutritional Concerns among Female International Volunteers Based on the Income and Development Status of Their Country of Service

**DOI:** 10.3390/ijerph19084846

**Published:** 2022-04-16

**Authors:** Megan J. Jensen, Katie N. Brown, Jennifer M. Turley, Marlene I. Graf, Jenna Dyckman, Andrew R. Creer, Susan Fullmer

**Affiliations:** 1 Department of Nutrition, Dietetics and Food Sciences, College of Agriculture and Applied Sciences, Utah State University, Logan, UT 84322, USA; megan.jensen@usu.edu (M.J.J.); marlene.graf@usu.edu (M.I.G.); 2 Department of Exercise & Nutrition Sciences, Moyes College of Education, Weber State University, Ogden, UT 84408, USA; jturley2@weber.edu; 3 Utah State University Extension, Utah State University, Logan, UT 84322, USA; jenna.dyckman@usu.edu; 4 Department of Exercise Science & Outdoor Recreation, College of Science, Utah Valley University, Orem, UT 84058, USA; andrew.creer@uvu.edu; 5 Department of Nutrition, Dietetics and Food Sciences, College of Life Sciences, Brigham Young University, Provo, UT 84602, USA; susan_fullmer@byu.edu

**Keywords:** female athlete triad, relative energy deficiency in sport, volunteers, amenorrhea, secondary amenorrhea, food insecurity, body satisfaction, weight loss, developing countries, low-income countries

## Abstract

This study aimed to determine the prevalence of female athlete triad risk factors among female international volunteers based on the development and income status of their country of service. A total of 2164 past volunteers completed a retrospective survey. Countries’ income and development statuses were coded using the respective annual United Nations World Economic Situations and Prospects reports. Independent t-tests, ANOVAs, and Pearson’s Chi-Squared tests were used to assess group differences; corresponding odds ratios were calculated. Volunteers in nondeveloped (OR = 2.25, CI = 1.85–2.75) and non-high-income (OR = 2.17, CI = 1.75–2.70) countries had over twice the odds of experiencing secondary amenorrhea. More volunteers who served in nondeveloped countries reported an increase in exercise while serving (*p* = 0.005). Those who served in a nondeveloped (OR = 1.52, CI = 1.16–1.98) or non-high-income (OR = 1.45, CI = 1.08–1.94) country had higher odds of weight loss. However, volunteers serving in nondeveloped (OR = 0.52, CI = 0.44–0.63) and non-high-income (OR = 0.50, CI = 0.4–0.61) countries were less likely to report food insecurity compared to those in developed and high-income countries. Bone mineral density was within the expected range regardless of income and development status. Female volunteers who served in nondeveloped and non-high-income countries experienced higher odds of secondary amenorrhea, which was likely influenced by an increase in exercise and higher odds of weight loss.

## 1. Introduction

The Church of Jesus Christ of Latter-Day Saints has a voluntary international missionary program. Men serve for 24 months, while women serve for 18 months. Though this study is specifically about volunteers for the Church of Jesus Christ of Latter-Day Saints, similar populations include individuals who serve in the Peace Corps, those who travel to other countries to teach a language, individuals in study abroad programs, and other international service-oriented programs. Currently, there are more than 53,000 volunteers serving in 404 missions (regions) throughout the world for the Church of Jesus Christ of Latter-day Saints; more than 22,000 of these have been reported to be women [1,2]. Volunteers are assigned to serve in dozens of countries throughout the world.

Depending on where a volunteer serves, they may be required to learn a new language and/or adapt to a new culture. With this comes a variety of potential lifestyle changes. For example, volunteers may walk and/or bike for transportation. This increases their physical activity and energy expenditure, thereby increasing the number of calories they need to maintain their weight. Volunteers may also have one or two meals per day provided by church members. This is typically a shared meal in members’ homes but the frequency and setting vary greatly depending on the concentration of members in the area and their ability to feed missionary volunteers [3]. Those who serve outside their home country will likely encounter foods and flavors that are unfamiliar to them. Many volunteers also report skipping meals on occasion because of a busy schedule or unexpected changes in their schedule. Therefore, most volunteers experience at least minor changes in their dietary patterns and intake while serving. Changes in dietary intake and exercise can put volunteers at risk for low energy availability (EA). Other factors can contribute to risk of low EA, such as cognitive dietary restraint, or the purposeful restraint of calories to control weight [4]. This is closely related to dieting behaviors, disordered eating, and body dissatisfaction [4].

The potential risk of low EA is concerning for female volunteers due to potential impacts on menstrual and bone health. This combination of low EA, menstrual irregularity, and low bone mineral density (BMD) is often seen in female athletes and is termed the Female Athlete Triad (Triad) [5]. The Triad is a female-specific syndrome that is part of Relative Energy Deficiency in Sport (RED-S) [6]. As not all aspects of RED-S were assessed in this study, the term Triad will be used. The original project by Friere et al. compared Triad risk factors between female volunteers and nonvolunteers of the same age [3]. This project reported that 30% of female volunteers experienced secondary amenorrhea while serving. It was also found that volunteers had increased odds of secondary amenorrhea compared to nonvolunteers (OR = 2.17, CI = 1.75–2.62) [3]. The prevalence of secondary amenorrhea among volunteers in the project by Friere et al. was similar to the rate reported among female athletes (20.9–36%) [5,7,8] and female Peace Corps members in Madagascar (27.7%) [9].

There has been no previous research done on nutritional risks in this specific population prior to Friere et al. [3]. Though Friere et al. determined the overall odds of developing amenorrhea in the volunteer population, this study aims to further analyze the data by income and development status of the country of service [3]. Previous research suggests that dietary intake may vary significantly from one country to another [10] and may be influenced by income status of the country [11]. Due to significant changes in lifestyle and culture that come with volunteer service, as well as expected shifts in diet and exercise habits, determining differences in nutritional risk based on the country may be helpful to decrease the severity of nutritional risks Through further analysis of the data used in the Friere et al. project [3], the purpose of this study was to determine whether differences in income and development status of the country of service impacted Triad risk of female volunteers.

## 2. Materials and Methods

### 2.1. Data Collection

The methods for this research project are published in detail in Friere et al. This study is a further analysis of the results found there [3]. This study focused on the participants from Friere et al. who were volunteers (*n* = 2164). Participants were college-aged women ages 21–26 years attending Brigham Young University (BYU), BYU-Idaho, Utah Valley University, Utah State University, the University of Idaho, and Weber State University. Participants were asked to complete an online survey that assessed their health during their volunteer missionary service (typically 18 months), and their health at the time they completed the survey. Participants could also opt to complete a retrospective online Diet History Questionnaire III (DHQ III) [12] and/or have a Dual-Energy X-ray Absorptiometry (DXA) scan to assess BMD.

### 2.2. Triad Factors

The survey assessed various factors related to the Triad using a variety of validated tools when available. Participants were asked to report their height, weight (lowest & highest while serving), weight changes (gained weight, lost weight, gained and lost weight, or weight maintenance), and weight satisfaction (ranging from very dissatisfied to very satisfied). Participant height and weight were used to calculate their body mass index (BMI). Participant weight satisfaction was categorized as dissatisfied with their weight (very dissatisfied or somewhat dissatisfied) or not dissatisfied with their weight (very satisfied, somewhat satisfied, or neutral). Volunteers who were unsure if they experienced any weight changes were excluded from the analysis.

Participants reported the comparison of their physical activity during the time of their voluntary service to their preservice physical activity (more than, less than, no changes). Volunteers’ food insecurity was assessed using the National Center for Health Statistics’ Six-Item Food Insecurity Scale [13]. The DHQ III, a validated tool by the National Cancer Institute, estimated volunteers’ usual average intake of a variety of foods, including macronutrients, micronutrients, water, and food groups [12]. The Healthy Eating Index (HEI) was used to assess diet quality by comparing volunteers’ dietary intake to the Dietary Guidelines for Americans [14,15]. Menstrual health was assessed using questions from the low energy availability in females questionnaire (LEAF-Q) [16].

### 2.3. Country Classifications

Volunteers’ country of service was categorized based on the world region, development status, and income level. Data were coded based on information from the Country Classifications Statistical Annex in the United Nation’s World Economic Situation and Prospects (WESP) report, which is released annually [17]. The WESP report that corresponded to the year the volunteer started service was used to code the data. For example, the 2017 WESP was used to code data for the volunteers who started their service in 2017, while the 2018 WESP was used for those who started in 2018. This enabled the data to reflect any changes to country classifications that may have occurred during the project period years [17]. Countries were categorized into “developed” or “nondeveloped” (developing countries and countries whose economies were in transition) and “high-income” and “non-high-income” (low-income, lower middle-income, upper middle-income) [14]. A separate category of “heavily indebted” countries was also included; this category includes the countries with the highest levels of poverty and debt in the world [17]. Most of these countries are in Africa and the Middle East [17].

World region of the service country was coded from 1–19 and included every region in the WESP [17]. Through 2016, the United States, Canada, Japan, Australia, and New Zealand were labeled as “Other Developed Countries”. Starting in 2017, the WESP classified these countries as “North America” (the United States and Canada) and “Developed Asia and Pacific” (Japan, Australia, and New Zealand) [17]. For clarity, these countries were coded as “North America” and “Developed Asia and Pacific” for all years of the project. Countries in the region of “New European Union Member States” were renamed by the WESP to the “European Union 13” in 2017 [17]. For simplification, these are labeled as “New EU/EU-13” in the analyses.

A few volunteers served in regions that included multiple countries with differing development and income statuses. For example, the Baltic Region included Estonia, Latvia, Belarus and Lithuania. Regions that included countries with differing income and development categories were coded as “Multiple Countries Differing Statuses” and were removed from the final analysis. Similarly, for the few volunteers who served in multiple regions, their region was coded as “Multiple Mission Location” and were removed from analysis. However, it is important to note that volunteers who served in regions that included multiple countries were not excluded from the final analysis if all of the countries in the region had the same development and income statuses (e.g., Czech/Slovak Region).

If any country or region was not included in the WESP, or if it was only included in some categories related to lack of information about that country, then any empty category was coded as “Insufficient Data” and was excluded from analysis. Similarly, if participants did not include the country of service and the year they began their service, they were coded as “Incomplete Survey” and were excluded from analysis.

### 2.4. Statistical Analysis

Data were analyzed via IBM SPSS (SPSS version 26, SPSS Inc., Chicago, IL, USA). Means and standard deviations were calculated for continuous variables. Categorical data were summarized using frequencies and percent of total. When comparing outcome variables based on development or income status, independent t-tests and analysis of variance (ANOVA) tests were used for continuous variables, and Pearson’s Chi-Squared was used for categorical variables. Odds ratios (OR) and 95% confidence intervals (CI) were used to assess the odds of various health outcomes based on the income level or development status of the country of service. Significance was set at *p* < 0.05.

## 3. Results

### 3.1. Participant Characteristics

**Figure 1:**
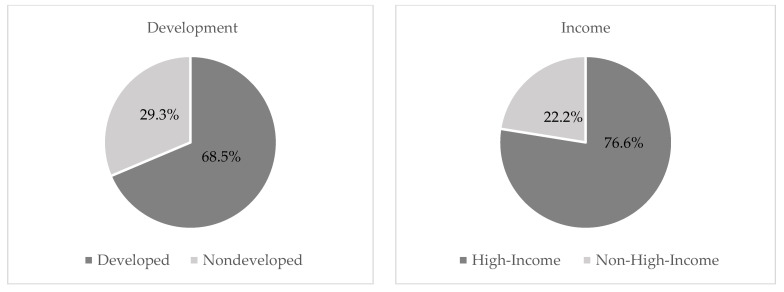
Volunteers’ division of income and development statuses of country of service.

Of the 2164 volunteers, 51% served in the United States, and 62.3% served in a major developed economy. Major developed economies (also known as the G7 economies) include the United States, the United Kingdom, Canada, France, Germany, Italy, and Japan [17]. Most volunteers (68.5%) served in developed countries, 29.3% in nondeveloped countries, and 2% in countries with transitioning economies. The majority of volunteers (76.6%) served in high-income countries, 16.1% served in upper-middle income, 5.9% served in lower-middle income, and 0.2% served in low-income countries. Only 0.2% of volunteers served in heavily indebted countries, and 0.8% served in least-developed countries. Data from volunteers who served in regions that included countries that differed in income and development status (1.7%) were not included in our final analysis. Due to small numbers of volunteers in some geographical regions, there was insufficient power to assess differences based on geographical region. The percentages of volunteers serving in high-income versus non-high-income and developed versus nondeveloped countries are displayed in Figure 1.

### 3.2. Odds of Triad Risk Factors Based on Country of Service’s Income and Development Status

**Figure 2:**
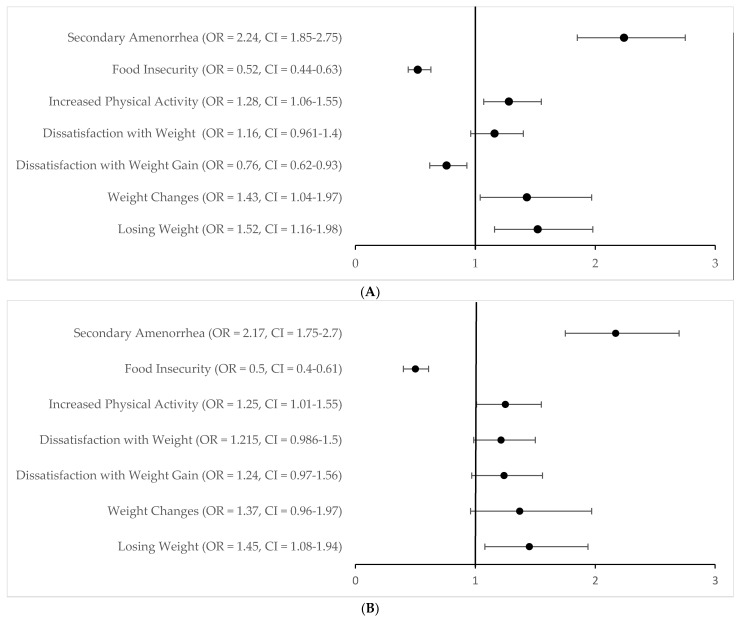
Volunteers’ odds of secondary amenorrhea and related factors when serving in nondeveloped (**A**) and non-high-income (**B**) countries.

As seen in Figure 2, volunteers who served in nondeveloped, and non-high-income countries had higher odds of secondary amenorrhea and had decreased odds of food insecurity in both cases. Volunteers who served in non-high-income countries also had increased physical activity during their volunteer service compared to prior to their volunteer service, had higher odds of weight changes, higher odds of weight loss, and lower odds of being dissatisfied with weight gain during their missionary service. Further details regarding the prevalence of Triad risk factors are described in subsequent sections.

### 3.3. Secondary Amenorrhea

The prevalence of secondary amenorrhea in the data sample was higher among volunteers who served in nondeveloped countries versus developed countries (41.98 vs. 24.3%, *p* < 0.001). The same trend was seen among volunteers who served in non-high-income countries (43.6 vs. 26.2%, *p* < 0.001). Volunteers who served in nondeveloped (OR = 2.24, CI = 1.85–2.75) and non-high-income countries (OR =2.17, CI = 1.75–2.70) had over twice the odds of experiencing secondary amenorrhea than their counterparts who served in developed and high-income countries.

### 3.4. Food Insecurity

Interestingly, more volunteers who served in developed or high-income countries reported more food insecurity than those who served in nondeveloped or non-high-income countries (Development: 61.5 vs. 45.6%, Income: 60.5 vs. 43.3%; *p* < 0.001). Serving in a nondeveloped (OR = 0.52, CI = 0.44–0.63) or non-high-income (OR = 0.50, CI = 0.4–0.61) country seemed to provide a protective effect against experiencing food insecurity.

### 3.5. Physical Activity

Data on frequency of physical activity versus the development and income status of country of service were also calculated. It was found that 43.5% of volunteers who served in nondeveloped countries exercised more than they did preservice versus 38.2% of volunteers who served in developed countries (*p* = 0.005). Similar percentages were seen in volunteers who served in non-high-income countries versus high-income countries, though the differences were not statistically significant (43.4 vs. 39%; *p* = 0.057). The odds of a volunteer reporting an increase in exercise in a nondeveloped country were higher than that of a developed country (OR = 1.28, CI = 1.06–1.55).

### 3.6. Changes and Satisfaction with Weight

There was a higher prevalence of weight gain among volunteers who served in developed countries compared to those who served in nondeveloped countries (41 vs. 32.1%, *p* < 0.001), whereas volunteers serving in nondeveloped countries had lower rates of experiencing both weight gain and loss during their service (31.3 vs. 38.6%, *p* < 0.001). As seen in Figure 2, volunteers who served in non-high-income and nondeveloped countries had higher odds of losing weight than those who served in high-income (OR = 1.45, CI 1.08–1.94) and developed countries (OR = 1.52, CI = 1.16–1.98). The odds of a volunteer experiencing weight changes while serving in a nondeveloped country was 1.43 (CI = 1.04–1.97). Similar patterns were seen for weight changes when making comparisons based on the income of the country (Weight Gain: Developed = 40.2%, Nondeveloped = 33%; Weight Gain/Loss: High-Income = 32%, Low-Income = 27.2% *p* = 0.026). However, volunteers in high-income countries were not at higher odds for weight changes (OR = 0.73, CI = 0.51–1.04).

### 3.7. Weight Satisfaction

Prevalence of weight dissatisfaction was higher among volunteers who gained weight during their service compared to volunteers who lost weight during their service (79.5 vs. 25.1%, *p* < 0.0001). As seen in Table 1, a high percentage of volunteers who maintained their weight during service experienced weight satisfaction. Volunteers who maintained weight while serving had over six times the odds of being satisfied with their weight compared to those who experienced weight changes (gained weight, lost weight, gained and lost weight) (OR = 6.34, CI = 4.55–8.84). There was not an increase in odds of weight dissatisfaction for volunteers serving in high income (OR = 1.22, CI = 0.99–1.50), or developed (OR = 1.16, CI = 0.96–1.40) countries. Furthermore, volunteers who gained weight and were dissatisfied about their weight gain were more likely to serve in developed countries (OR = 1.32, CI = 1.08–1.62). Increased odds of gaining weight and having weight dissatisfaction were not observed in volunteers in non-high-income countries (OR = 1.24, CI = 0.97–1.56).

### 3.8. Dietary History Questionnaire

Results from the DHQ III indicated that volunteers who served in nondeveloped and non-high-income countries reported eating more calories (2210.73 ± 942.59 vs. 1900.14 ± 764.44, *p* = 0.017; 2247.32 ± 943.89 vs. 1916.04 ± 777.05, *p* = 0.014), protein (g) (84.84 ± 37.75 vs. 73.29 ± 29.72, *p* = 0.016; 86.88 ± 38.54 vs. 73.48 ± 29.43, *p* = 0.010), animal protein (g) (55.77 ± 27.71 vs. 46.89 ± 20.28, *p* = 0.018; 57.56 ± 27.98 vs. 46.80 ± 19.82, *p* = 0.013), added sugars (g) (89.09 ± 51.99 vs. 73.77 ± 33.42, *p* = 0.025; 89.00 ± 48.19 vs. 75.79 ± 38.63, *p* = 0.049), total sugars (g) (136.34 ± 68.51 vs. 108.51 ± 46.32, *p* = 0.002; 134.17 ± 62.98 vs. 111.80 ± 51.58, *p* = 0.012), polyunsaturated fat (g) (16.85 ± 8.35 vs. 14.27 ± 6.75, *p* = 0.025; 17.17 ± 8.55 vs. 14.41 ± 6.87, *p* = 0.040), and total fruit (cups) (1.86 ± 1.19 vs. 1.31 ± 1.01, *p* < 0.001; 1.78 ± 1.12 vs. 1.39 ± 1.03, *p* = 0.025), respectively. Those who served in non-high-income countries also consumed more sodium (g) (3570.56 ± 1504.16 vs. 3115.37 ± 1290.87, *p* = 0.038). Those who served in nondeveloped countries consumed more total vegetables (cups) (1.89 ± 1.13 vs. 1.51 ± 0.96, *p* = 0.014), a higher percentage of calories from carbohydrates (54.61 ± 5.25 vs. 52.17 ± 5.31, *p* = 0.002), and a lower percentage of calories from fat (31.35 ± 4.03 vs. 33.77 ± 4.51, *p* < 0.001) compared to those who served in developed countries. See Supplementary Materials for more details regarding dietary intake.

### 3.9. Healthy Eating Index

Dietary data were further analyzed for differences in the HEI (Table 2 and Table 3). There was no difference in overall HEI score, indicating no difference in overall diet quality. Volunteers serving in nondeveloped and non-high-income countries had significantly lower HEI component scores for whole grains and dairy compared to those in high-income and developed countries. However, volunteers in non-high-income and nondeveloped countries had a higher HEI component score for saturated fat. Volunteers serving in nondeveloped countries also had a higher HEI component score for total fruit than those in developed countries.

### 3.10. DXA Results

There were no significant differences in BMD based on the income or development of the country of service. The average BMD for each participant was within the expected range for age (see Table 4).

## 4. Discussion

In this study, volunteers who served in nondeveloped and non-high-income countries had over twice the odds of experiencing secondary amenorrhea compared to those who served in developed and high-income countries.

Volunteers who served in nondeveloped countries had higher odds (OR = 1.28) of exercising more while serving compared to a preservice time period. In addition, volunteers in nondeveloped countries also had higher odds of experiencing weight changes while serving (OR = 1.43), and those in both nondeveloped or non-high-income had higher odds of weight loss (OR = 1.46 and 1.37). This creates a more accurate picture as to why higher odds of secondary amenorrhea were seen in the volunteers who served in non-high-income and nondeveloped countries. Though they reported eating more calories compared to their counterparts, they also reported higher rates of both exercise and weight loss, indicating they were likely not eating enough calories to compensate for the calories they were expending.

The correlation between exercise, weight loss, and secondary amenorrhea has been documented in previous research. It has been found that approximately 50% of women who exercise regularly have subtle menstrual disturbances, while 30% of women who exercise regularly have secondary amenorrhea [8,18]. Though rapid weight loss is a risk factor for secondary amenorrhea, this does not mean a person has to be emaciated to experience secondary amenorrhea. A 10–15% weight loss, even when someone is 90–110% of ideal body weight, is associated with secondary amenorrhea [19,20].

A surprising finding was that odds of experiencing food insecurity were lower for volunteers serving in nondeveloped and non-high-income countries compared to developed and high-income countries (OR = 0.52 and 0.50, respectively). There are many possible explanations for this as well. Volunteers serving in nondeveloped and non-high-income countries may have expected dramatic changes to their diets, so the actual change may not be seen as a surprise to them. Many of the citizens of those countries may have been eating similarly to them, with volunteers often being fed more frequently by church members. Therefore, compared to others living in these countries, volunteers likely had either similar or greater access to food. Findings from a previous study among college students suggest that perceptions of what is normal may influence perceptions of food security; college students who viewed skipping meals as “normal” may not have considered themselves food insecure [21]. Knowing this, cultural perceptions could impact how volunteers answered the food insecurity questions in the survey based on what was “normal” in their country of service.

It is also important to note that though the volunteers serving in nondeveloped and non-high-income countries did not report higher levels of food insecurity, this does not mean that they consumed the recommended amount of nutrients, particularly for their activity level. Contrary to what was expected, volunteers who served in non-high-income and nondeveloped countries reported eating more during service compared to their counterparts who served in developed and high-income countries. They ate significantly more calories, total protein, animal protein, added sugars, total sugars, polyunsaturated fats, and fruit compared to their counterparts. Volunteers who served in nondeveloped countries also ate significantly more vegetables, percent of daily calories from fat, and percent of daily calories from carbohydrates.

There were no differences in overall HEI score, indicating no difference in overall diet quality based on the country’s income or development status. However, there were some notable exceptions. Volunteers serving in non-high-income and nondeveloped countries ate significantly fewer whole grains and dairy products, despite reporting a higher calorie intake, when compared to those in high-income and developed countries.

Differences in dairy intake could partially be explained by the differences in dairy intake by world region. Regions that tend to consume more dairy products include North America, Europe, and parts of South America, while areas that consume less include some parts of South America, Africa and Southeast Asia [22]. As these three low-dairy consuming areas of the world have a higher density of countries that are nondeveloped and non-high-income, this could explain why significant differences were seen [17,22]. Dairy consumption is especially low in Africa and Southeast Asia, and these regions have the highest rates of lactose malabsorption and intolerance in the world [23].

Differences in whole grain intake could be due to the amount of attention whole and “ancient” grains are getting in the media in developed and high-income countries. The Whole Grain Council reports that 58% of consumers are actively seeking more whole grains in their diet, 61% recognize that whole grains are beneficial for digestion, and that younger generations are more likely to purchase whole grain baked goods [24]. These reports are coming from mostly high-income and/or developed countries, such as the United States, Italy, Australia, and Singapore. Whole grains are actively getting more attention throughout the world, but only 29% of whole grain products can be found outside of the United States. Therefore, these products are most likely seen and purchased in high-income and developed countries [24].

Despite eating less dairy and fewer whole grains, volunteers in nondeveloped and non-high-income countries ate more saturated fat. A 2014 global study about saturated fat intake found that countries in Central Europe, Russia, coastal and central Africa, and Southeast Asia had the highest percent of energy from saturated fat intake [25]. As mentioned previously, these are the regions with higher concentrations of nondeveloped and non-high-income countries, which would partially explain high intake among voluneers serving in those areas. The increasing Westernization of diets in nondeveloped and non-high-income countries, as well as the increased intake of red meats and palm oil, may also have influenced these findings [25].

Data on weight satisfaction based on development and income of the country were also assessed. There was no difference in overall weight satisfaction between country of service when income or development status was factored in. However, more volunteers who maintained their weight while serving had higher odds (OR = 6.34) of being satisfied with their weight, regardless of development and income status of the country. Volunteers who reported dissatisfaction about their weight had lower odds of serving in nondeveloped countries (OR = 0.76), while those who experienced weight loss and were dissatisfied about it did not have different odds based on the development and income status of the country. Volunteers who served in developed and high-income countries had higher rates of weight gain (~41%) than those in nondeveloped and non-high-income countries (~32%), which may explain greater dissatisfaction with their weight. Another factor that may have influenced this finding is that volunteers serving in nondeveloped and non-high-income countries may have expected to experience some weight loss (based on recommendations [26] and reports from others who had served in various capacities in nondeveloped countries [27]), so they may not have been concerned when this did occur.

This study is limited in that the data were both self-reported and retrospective. In addition, the internal standardized DHQ III instructions asked participants to report what they ate within the past year. This discrepancy may have caused confusion despite being instructed in the survey to report what they ate during the period of time that they were a volunteer.

## 5. Conclusions

Volunteers who served in nondeveloped and non-high-income countries had over twice the odds of experiencing secondary amenorrhea compared to their counterparts who served in high-income and developed countries. Volunteers in non-high-income and nondeveloped countries also reported higher incidences of exercise, which likely influenced odds of secondary amenorrhea. Though the volunteers in non-high-income and nondeveloped countries reported eating significantly more calories than those in developed and high-income countries, their increased energy expenditure seemed to offset this, potentially causing the increased odds of weight loss.

One unexpected outcome of this study was that volunteers in nondeveloped and non-high-income countries reported more food security than their counterparts in developed and high-income countries. There are a number of possible reasons for this, including cultural differences and interpretations of adequate diet quality and quantity.

Leaders of volunteers could consider scheduling annual or biannual wellness check-ups where dietary intake and menstrual health of volunteers could be assessed by qualified medical professionals. Preservice training of volunteers could also include more education about the risk factors and negative health effects associated with secondary amenorrhea and how to seek help from medical professionals. This information could be particularly helpful for volunteers who are assigned to nondeveloped and non-high-income countries, because their risk level may be higher.

Future studies could consider assessing the risks among volunteers serving in individual countries, as there can be development and income differences within a single country. Because there are often differing income and development levels within different regions of an individual country, data based on individual region would be more accurate to determine Triad risk in female volunteers.

## Figures and Tables

**Table 1:** Weight changes and satisfaction during volunteer service (*n* = 2058 ^a^).

	Dissatisfied (*n* = 1187)	Satisfied (*n* = 563)	Neutral (*n* = 307)	*p*-Value
	*n* (%)	*n* (%)	*n* (%)	<0.001
Gained Weight	652 (54.9)	81 (14.40)	86 (28.01)	
Gained/Lost Weight	417 (35.13)	182 (32.32)	129 (42.02)	
Lost Weight	69 (5.81)	155 (27.53)	51 (16.6)	
Maintained Weight	48 (4.04)	144 (25.60)	39 (12.70)	

^a^ Four participants selected “Don’t Know”; Not all participants answered all questions.

**Table 2:** HEI scores based on country development category (*n* = 207).

	Developed (*n* = 134)	Nondeveloped (*n* = 73)	*p*-Value
	Mean ± SD	Mean ± SD	
HEI Total Score	62.36 ± 9.27	63.96 ± 8.31	0.22
**HEI Adequacy Component Scores**			
Total Fruits	3.52 ± 1.45	4.02 ± 1.23	0.01
Whole Fruits	4.29 ± 1.26	4.51 ± 0.91	0.15
Total Vegetables	3.68 ± 1.04	3.79 ± 1.08	0.47
Greens and Beans	3.85 ± 1.49	3.42 ± 1.85	0.09
Whole Grains	4.10 ± 2.06	3.32 ± 2.01	0.01
Dairy	6.60 ± 2.41	5.53 ± 2.51	0.003
Total Protein Foods	4.71 ± 0.60	4.74 ± 0.58	0.79
Seafood and Plant Proteins	3.98 ± 1.34	3.82 ± 1.48	0.44
Fatty Acids	4.49 ± 2.75	5.50 ± 2.78	0.12
**Moderation HEI Component Scores**			
Refined Grains	6.93 ± 2.80	7.38 ± 2.63	0.26
Sodium	3.98 ± 2.14	4.56 ± 2.27	0.07
Added Sugars	6.32 ± 2.21	6.02 ± 2.74	0.43
Saturated Fats	5.92 ± 2.55	7.34 ± 2.23	<0.001

**Table 3:** HEI scores based on country income category (*n* = 203).

	High-Income (*n* = 152)	Non-High-Income (*n* = 51)	*p*-Value
	Mean ± SD	Mean ± SD	
HEI Total Score	62.85 ± 9.08	63.37 ± 8.77	0.726
**HEI Adequacy Component Scores**			
Total Fruits	3.63 ± 1.43	3.88 ± 1.33	0.280
Whole Fruits	4.35 ± 1.22	4.40 ± 0.97	0.799
Total Vegetables	3.74 ± 1.03	3.67 ± 1.13	0.704
Greens and Beans	3.79 ±1.51	3.61 ± 1.85	0.526
Whole Grains	4.08 ± 2.06	3.17 ± 2.03	0.007
Dairy	6.42 ± 2.49	5.58 ± 2.38	0.036
Total Protein Foods	4.70 ± 0.63	4.77 ± 0.48	0.472
Seafood and Plant Proteins	3.94 ± 1.36	3.93 ± 1.44	0.967
Fatty Acids	4.64 ± 2.77	5.51 ± 2.77	0.053
**Moderation HEI Component Scores**			
Refined Grains	7.05 ± 2.72	7.24 ± 2.82	0.658
Sodium	4.10 ± 2.21	4.38 ± 2.20	0.427
Added Sugars	6.23 ± 2.33	6.05 ± 2.67	0.664
Saturated Fats	6.18 ± 2.56	7.15 ± 2.25	0.016

**Table 4:** Average BMD and Z-Scores at all sites by development and income status of country of service (*n* = 401).

	High-Income (*n* = 294)	Not High-Income (*n* = 107)	*p*-Value
	Mean ± SD	Mean ± SD	
Neck Mean Z-Score	0.18 ± 0.94	0.216 ± 0.95	0.79
Neck Low BMD (g/cm^2^)	0.00 ± 0.06	0.00 ± 0.00	0.55
Troch Low BMD(g/cm^2^)	0.04 ± 0.19	0.04 ± 0.19	0.99
Troch Mean Z-Score	−0.23 ± 1.034	−0.219 ± 1.03	0.95
Total Mean Z-Score	0.48 ± 0.99	0.464 ± 0.98	0.89
Total Low BMD (g/cm^2^)	0.00 ± 0.59	0.00 ± 0.00	0.55
L1-L4 Z-Score	0.28 ± 0.92	0.377 ± 0.97	0.36
L1-L4 Low BMD (g/cm^2^)	0.00 ± 0.06	0.00 ± 0.00	0.55
	**Developed** **(*n* = 264)**	**Nondeveloped** **(*n* = 146)**	***p*-Value**
Neck Mean Z Score	0.16 ± 0.91	0.32 ± 1.01	0.09
Neck Low BMD (g/cm^2^)	0.00 ± 0.62	0.00 ± 0.00	0.46
Troch Low BMD(g/cm^2^)	0.04 ± 0.20	0.03 ± 0.17	0.47
Troch Mean Z Score	−0.26 ± 1.01	−0.16 ± 1.08	0.31
Total Mean Z Score	0.45 ± 0.97	0.57 ± 1.04	0.24
Total Low BMD (g/cm^2^)	0.00 ± 0.06	0.00 ± 0.00	0.46
L1-L4 Z Score	0.27 ± 0.92	0.41 ± 0.96	0.16
L1-L4 Low BMD (g/cm^2^)	0.00 ± 0.06	0.00 ± 0.00	0.46

## Data Availability

The data presented in this study are available on request from the corresponding author.

## Supplementary Materials

The following supporting information can be downloaded at: https://www.mdpi.com/article/10.3390/ijerph19084846/s1, Table S1: Participants’ Reported Consumption of Select Nutrients, Foods, and Food Groups (from DHQ III) Based on Country Income Category, Table S2: Participants’ Reported Consumption of Select Nutrients, Foods, and Food Groups (from DHQ III) Based on Country Development Category.
