# Supplementary material for: Nutritional Concerns among Female International Volunteers Based on the Income and Development Status of Their Country of Service
Source: Int J Environ Res Public Health. 2022 Apr 16;19(8):4846. doi: 10.3390/ijerph19084846 (PMC9026757; doi:10.3390/ijerph19084846)
Supplement: Supplementary file 1 [file ijerph-19-04846-s001.zip › ijerph-1655531-supplementary.pdf]

Supplementary Table S1. Participants' Reported Consumption of Select Nutrients, Foods, and Food Groups (from DHQ III) Based on Country Income Category

	**High-Income (n=152)**	**Non-High-Income (n=51)**	**P-Value**
**Nutrient**	Mean ± SD	Mean ± SD	
Calories (kcal)	1916.04 ± 777.05	2247.32 ± 943.89	0.014
Protein (g)	73.48 ± 29.43	86.88 ± 38.54	0.010
Animal Protein (g)	46.80 ± 19.82	57.56 ± 27.98	0.013
Vegetable Protein (g)	26.68 ± 12.49	29.32 ± 13.17	0.199
Total Fat (g)	71.47 ± 32.05	80.94 ± 38.02	0.083
Added Sugars (g)	75.79 ± 38.63	89.00 ± 48.19	0.049
Total Dietary Fiber (g)	20.39 ± 10.15	23.03 ± 10.82	0.115
Total Sugars (g)	111.80 ± 51.58	134.17 ± 62.98	0.012
Total Saturated Fat (g)	24.02 ± 11.25	26.55 ± 13.21	0.186
Total Monounsaturated Fat (g)	26.28 ± 12.24	29.12 ± 13.61	0.165
Total Polyunsaturated Fat (g)	14.41 ± 6.87	17.17 ± 8.55	0.040
Vitamin D (mcg)	4.81 ± 2.47	4.84 ± 3.37	0.955
Iron (mg)	15.59 ± 6.85	16.28 ± 7.22	0.537
Calcium (mg)	1015.46 ± 417.91	1038.52 ± 482.89	0.744
Vitamin B12 (mcg)	5.04 ± 2.52	4.99 ± 2.41	0.917
Vitamin B6 (mg)	2.34 ± 0.99	2.61 ± 1.17	0.105
Total Folate (mcg)	454.58 ± 194.45	479.39 ± 200.11	0.435
Phosphorus (mg)	1292.70 ± 511.57	1458.67 ± 649.65	0.063
Zinc (mg)	11.92 ± 5.19	12.46 ± 5.40	0.524
Sodium (mg)	3115.37 ± 1290.87	3570.56 ± 1504.16	0.038
Water (g)	3080.03 ± 1088.66	3599.57 ± 1042.48	0.003
Total Fruit (cups)	1.39 ± 1.03	1.78 ± 1.12	0.025
Total Vegetable (cups)	1.56 ± 0.95	1.88 ± 1.25	0.095
Legumes (cups)	0.15 ± 0.19	0.22 ± 0.25	0.084
Grains (oz)	5.98 ± 3.03	6.67 ± 3.90	0.195
Protein Foods (oz)	5.22 ± 2.69	6.01 ± 2.98	0.076
Total Dairy (cups)	1.64 ± 0.86	1.71 ± 1.22	0.625
Energy from Fat (%kcal)	33.29 ± 4.58	31.91 ± 4.13	0.059
Energy from CHO (%kcal)	52.73 ± 5.39	53.93 ± 5.44	0.170
Energy from Protein (%kcal)	15.74 ± 2.39	15.76 ± 2.47	0.961

Supplementary Table S2. Participants' Reported Consumption of Select Nutrients, Foods, and Food Groups (from DHQ III) Based on Country Development Category

	**Developed (n=152)** Mean ± SD	**Non-Developed (n=51)** Mean ± SD	**P-Value**
**Nutrient**			
Calories (kcal)	1900.14 ± 764.44	2210.73 ± 942.59	0.017
Protein (g)	73.29 ± 29.72	84.84 ± 37.75	0.016
Animal Protein (g)	46.89 ± 20.28	55.77 ± 27.71	0.018
Vegetable Protein (g)	26.41 ± 12.39	29.07 ± 12.99	0.148
Total Fat (g)	71.97 ± 32.18	78.16 ± 37.06	0.211
Added Sugars (g)	73.77 ± 33.42	89.09 ± 51.99	0.025
Total Dietary Fiber (g)	20.19 ± 10.29	22.63 ± 10.34	0.107
Total Sugars (g)	108.51 ± 46.32	136.34 ± 68.51	0.002
Total Saturated Fat (g)	24.31 ± 11.35	25.67 ± 13.10	0.438
Total Monounsaturated Fat (g)	26.52 ± 12.33	28.09 ± 13.29	0.396
Total Polyunsaturated Fat (g)	14.27 ± 6.75	16.85 ± 8.35	0.025
Vitamin D (mcg)	4.82 ± 2.51	5.24 ± 4.12	0.370
Iron (mg)	15.65 ± 6.99	15.94 ± 6.76	0.775
Calcium (mg)	1030.53 ± 423.06	1031.73 ± 521.35	0.986
Vitamin B12 (mcg)	5.13 ± 2.59	5.16 ± 3.47	0.942
Vitamin B6 (mg)	2.34 ± 1.01	2.53 ± 1.11	0.219
Total Folate (mcg)	455.15 ± 196.45	471.51 ± 194.29	0.566
Phosphorus (mg)	1301.84 ± 519.22	1422.67 ± 653.98	0.147
Zinc (mg)	12.09 ± 5.31	12.14 ± 5.39	0.951
Sodium (mg)	3120.46 ± 1311.69	3465.47 ± 1439.17	0.082
Water (g)	3016.78 ± 1097.56	3602.98 ± 1066.85	<0.001
Total Fruit (cups)	1.31 ± 1.01	1.86 ± 1.19	<0.001
Total Vegetable (cups)	1.51 ± 0.96	1.89 ± 1.13	0.014
Legumes (cups)	0.16 ± 0.19	0.18 ± 0.23	0.521
Grains (oz)	6.03 ± 3.12	6.43 ± 3.52	0.402
Protein Foods (oz)	5.19 ± 2.70	5.91 ± 2.93	0.079
Total Dairy (cups)	1.67 ± 0.86	1.74 ± 1.41	0.667
Energy from Fat (%kcal)	33.77 ± 4.51	31.35 ± 4.03	<0.001
Energy from CHO (%kcal)	52.17 ± 5.31	54.61 ± 5.25	0.002
Energy from Protein (%kcal)	15.80 ± 2.29	15.65 ± 2.59	0.660
